# Functional Biomaterials for Vascular Healing After Percutaneous Coronary Intervention: From Surface Engineering to Clinical Translation

**DOI:** 10.3390/ijms272110033

**Published:** 2026-11-09

**Authors:** Jae Seok Bae, Jeong Yoon Jang, Yun-Ho Cho, Yujin Shin, Hyo Jin Lee, Sung-Eun Park, Jong-Hwa Ahn

**Affiliations:** 1 Division of Cardiology, Department of Internal Medicine, Gyeongsang National University School of Medicine and Gyeongsang National University Changwon Hospital, Changwon 51472, Republic of Korea; 2 Department of Radiology, Gyeongsang National University School of Medicine and Gyeongsang National University Changwon Hospital, Changwon 51472, Republic of Korea

**Keywords:** biomaterials, percutaneous coronary intervention, vascular healing, drug-eluting stent, drug-coated balloon, bioresorbable scaffold, surface engineering, endothelialization, biomimetic materials

## Abstract

Percutaneous coronary intervention (PCI) creates a dynamic blood–material–tissue interface in which thrombosis, inflammation, smooth muscle cell proliferation, and endothelial recovery evolve simultaneously. This narrative review examines coronary biomaterials using a healing-centered translational framework that links material properties to molecular and cellular responses, tissue-level healing, measurable structural and functional phenotypes, and clinical outcomes. We review the evolution from bare-metal and drug-eluting stents to polymer-free systems, drug-coated balloons (DCBs), bioresorbable scaffolds, and bioactive or biomimetic surfaces. Particular emphasis is placed on strut geometry, polymer chemistry and degradation, drug-release kinetics, surface properties, corrosion behavior, endothelial barrier function, macrophage responses, thrombogenicity, and neointimal growth. Structural coverage on intravascular imaging is distinguished from functional endothelial recovery, which requires complementary biological or physiological assessment. Randomized evidence is interpreted at the level of the tested device, formulation, lesion, and procedural strategy; newer platforms are not assumed to confer class-wide superiority. Future progress will require multifunctional, healing-responsive platforms that integrate antithrombotic, pro-endothelial, immunomodulatory, and controlled-delivery functions while demonstrating mechanical durability, comparative clinical effectiveness, manufacturability, and cost-effective scalability.

## 1. Introduction

Percutaneous coronary intervention (PCI) restores coronary lumen dimensions, but its durable success also depends on the biological response of the treated vessel. Coronary devices interact with an injured, atherosclerotic arterial wall rather than an inert conduit. The introduction of drug-eluting stents (DES) established that local pharmacological modification of this response could substantially reduce restenosis: the RAVEL trial demonstrated the efficacy of sirolimus delivery in suppressing neointimal proliferation relative to a conventional metallic stent [1]. Subsequent human pathology studies revealed an accompanying challenge: strong inhibition of tissue growth could coexist with delayed arterial healing and late thrombotic vulnerability [2]. These findings place vascular repair at the center of coronary biomaterial design.

Contemporary DESs incorporate improvements in metallic platforms, strut geometry, polymer coatings, and drug delivery. Human autopsy comparisons have documented more favorable healing characteristics with second-generation everolimus-eluting stents than with first-generation sirolimus- and paclitaxel-eluting devices [3]. Nevertheless, long-term clinical analyses show that adverse events continue beyond the first year after implantation [4]. The engineering objective therefore extends beyond maximizing acute lumen gain or minimizing neointimal thickness. It includes achieving a stable blood–material interface and sustained vascular compatibility while retaining sufficient mechanical support and antirestenotic efficacy.

In this review, functional coronary biomaterials are defined as materials whose composition, architecture, surface chemistry, drug-delivery properties, or degradation behavior are designed to influence vascular responses. This definition encompasses durable and biodegradable DES coatings, polymer-free drug reservoirs, drug-coated balloon (DCB) formulations, bioresorbable scaffolds, and bioactive or biomimetic surface modifications. These approaches intervene at different points in the injury–repair cascade. For example, CD31-mimetic stent coatings have promoted endothelialization while reducing inflammation and neointimal development in preclinical experiments [5]. Such findings illustrate the possibility of combining biological functions within a single platform, although clinical efficacy must be established separately.

Recent randomized evidence also demonstrates why biological plausibility cannot substitute for clinical validation. The AGENT IDE trial supported paclitaxel-coated balloon treatment over uncoated balloon angioplasty for coronary in-stent restenosis [6]. In contrast, the three-year REC-CAGEFREE I results showed a higher device-oriented composite event rate with a DCB strategy incorporating rescue stenting than with up-front DES implantation for de novo coronary disease [7]. Similarly, the seven-year COMPARE-ABSORB results did not establish the anticipated post-resorption superiority of the studied polymeric scaffold over metallic everolimus-eluting stents [8]. These observations argue for evaluating each material system within its tested device, indication, and procedural context.

**Figure 1:**
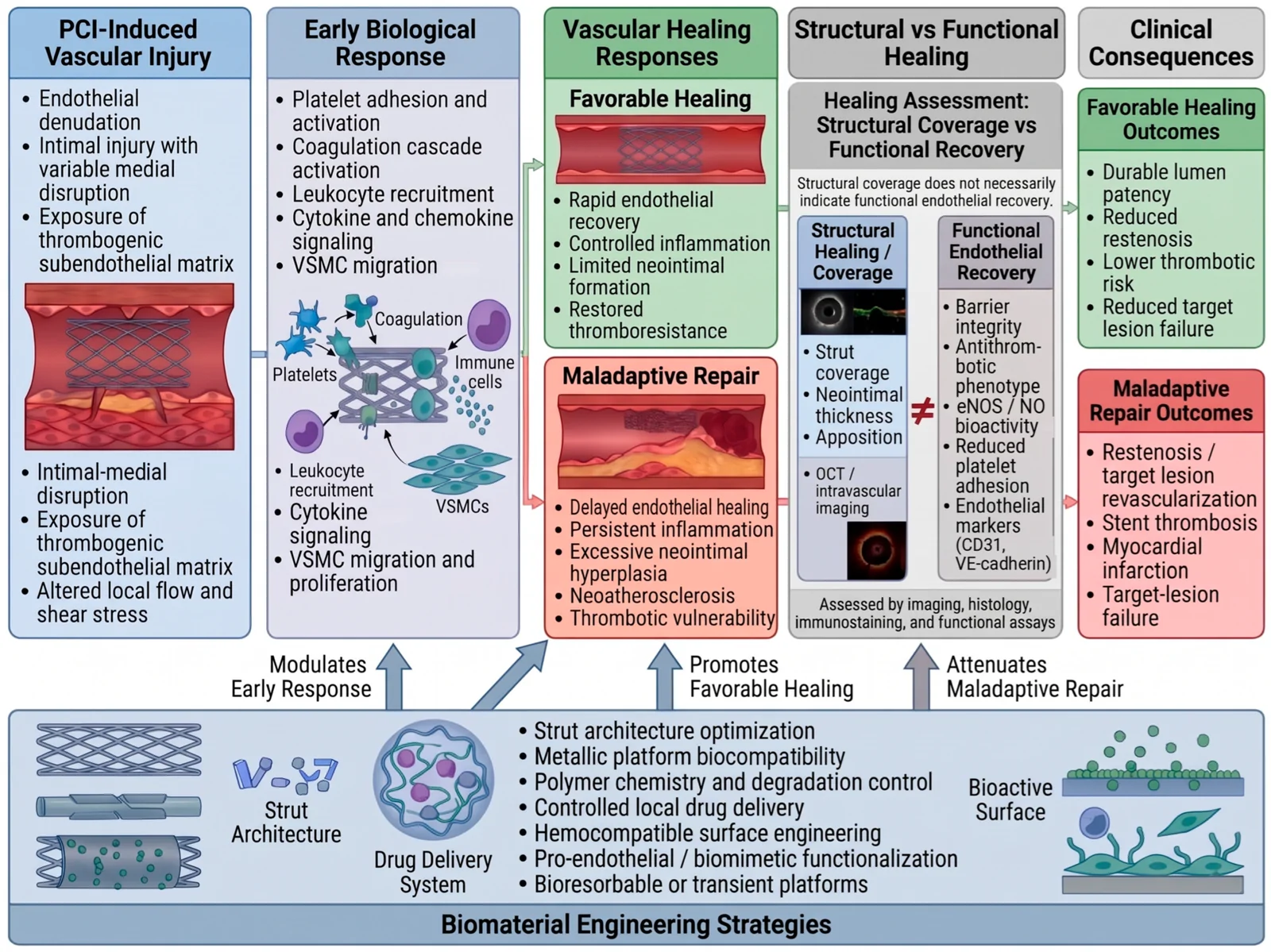
Biological cascade of vascular healing after percutaneous coronary intervention and targets for biomaterial engineering. PCI-induced vascular injury activates platelets, coagulation, inflammatory signaling, leukocyte recruitment, and VSMC responses. Favorable healing is characterized by functional endothelial recovery, controlled inflammation, limited neointimal formation, and restored thromboresistance, whereas maladaptive repair includes delayed endothelial recovery, persistent inflammation, excessive neointimal hyperplasia, neoatherosclerosis, and thrombotic vulnerability. Structural healing assessed by OCT is explicitly distinguished from functional endothelial recovery assessed by molecular, histological, and physiological measures. In the schematic, blue indicates the initial vascular injury and early biological response, green indicates favorable vascular healing, red indicates maladaptive repair, and the lower colored boxes represent the corresponding biomaterial engineering strategies.

**Figure 2:**
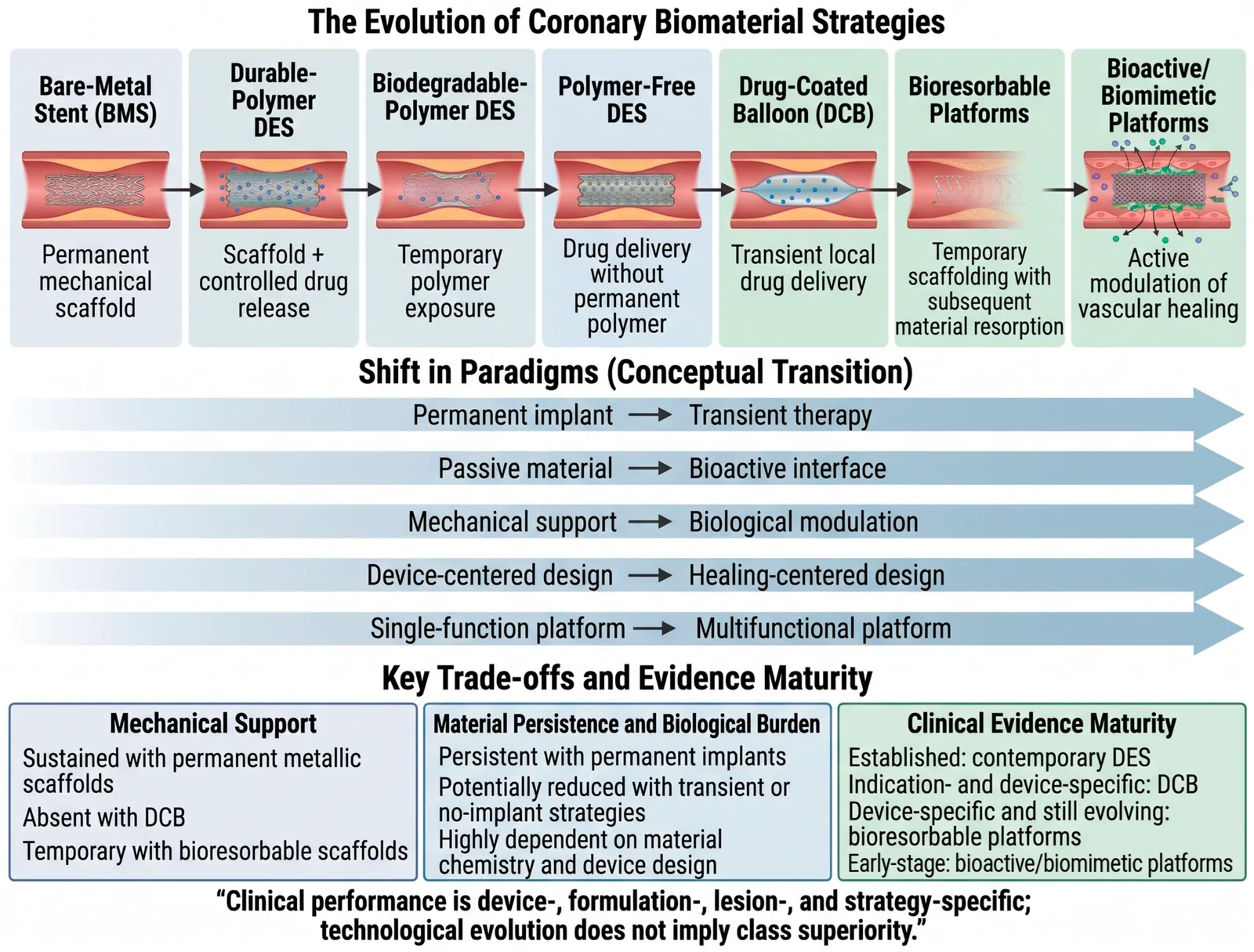
Evolution of coronary biomaterial strategies and their principal translational trade-offs. The schematic summarizes the transition from bare-metal and polymer-based DESs toward polymer-free drug delivery, DCBs, bioresorbable platforms, and bioactive or biomimetic interfaces. The arrows indicate the conceptual evolution of coronary biomaterial strategies rather than a hierarchy of clinical superiority. Colors are used to distinguish the major device categories and translational trade-offs across the different biomaterial platforms. Contemporary DESs have the most mature comparative evidence; DCB outcomes remain indication-, formulation-, and strategy-specific; and evidence for bioresorbable and emerging bioactive platforms remains device-specific and evolving.

**Figure 3:**
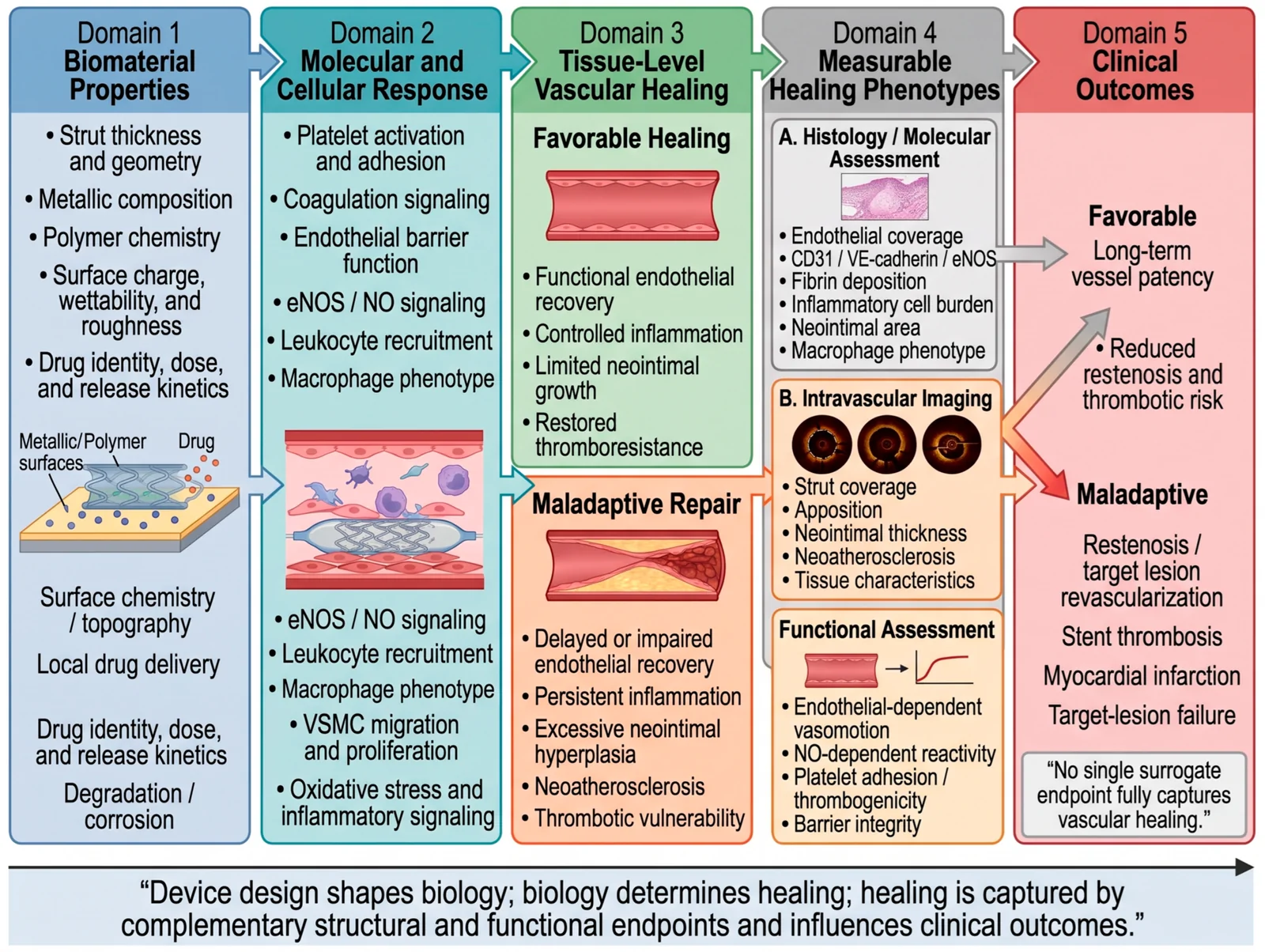
Healing-centered translational framework linking biomaterial properties to molecular/cellular responses, tissue-level vascular healing, measurable phenotypes, and clinical outcomes. Functional endothelial recovery is distinguished from structural coverage and is represented by barrier integrity, eNOS/NO signaling, antithrombotic phenotype, low platelet adhesion, and physiological vasomotion. Histology/molecular assessment, OCT, and functional testing provide complementary but noninterchangeable information; no single surrogate fully captures vascular healing. Colors are used to distinguish the five domains of the translational framework, including biomaterial properties, molecular and cellular responses, tissue-level vascular healing, measurable healing phenotypes, and clinical outcomes.

**Figure 4:**
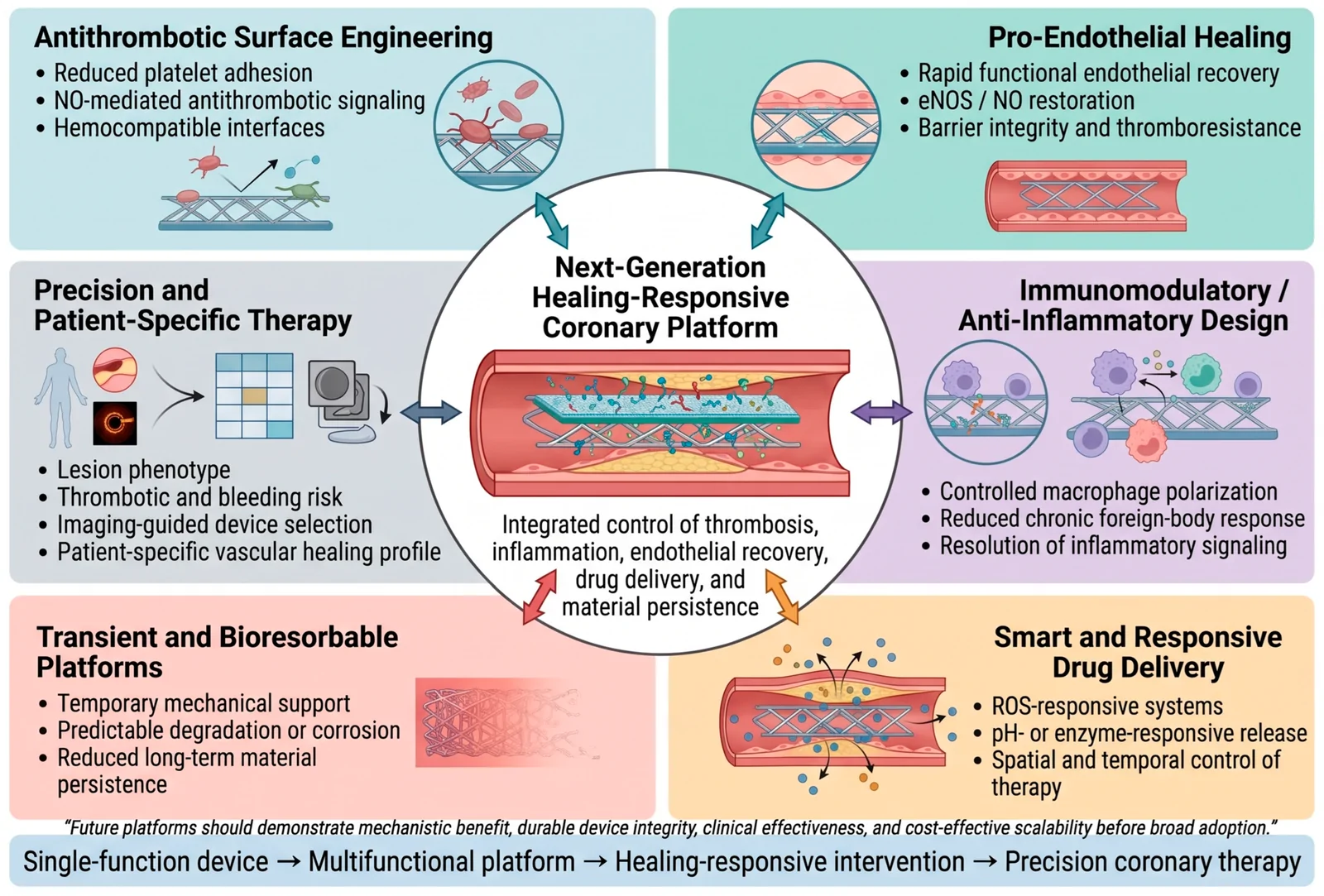
Future directions for next-generation healing-responsive coronary platforms. The framework integrates antithrombotic surface engineering, pro-endothelial recovery, immunomodulatory design, smart or responsive drug delivery, transient/bioresorbable support, and precision patient- and lesion-specific selection. These concepts remain at different stages of evidence maturity. Future platforms should demonstrate retained function after manufacturing and deployment, mechanistic benefit, comparative clinical effectiveness, and scalable cost-effectiveness before broad adoption. Colors are used to distinguish the major functional modules and translational priorities within the proposed healing-responsive coronary platform.

The central premise of this narrative review is that coronary biomaterials should be evaluated through a multilevel healing framework rather than by material class or device chronology alone. Unlike recent reviews that primarily summarize individual DES, DCB, resorbable, or surface-engineering technologies [9,10,11,12,13,14,15,16,17,18], we explicitly connect (i) material and surface properties, (ii) molecular and cellular responses, (iii) favorable versus maladaptive tissue repair, (iv) measurable structural and functional healing phenotypes, and (v) clinical outcomes. This hierarchy separates mechanistic plausibility from clinical evidence maturity and emphasizes that structural coverage is not equivalent to functional endothelial recovery. Section 2 defines the biological and operational healing targets, Section 3 outlines the evolution of coronary biomaterial strategies, and Section 4, Section 5, Section 6 and Section 7 critically examine DESs, DCBs, bioresorbable scaffolds, and bioactive surfaces. Section 8, Section 9 and Section 10 then integrate imaging, clinical outcomes, economics, evidence standards, and future development. Figure 1, Figure 2, Figure 3 and Figure 4 visualize the injury–repair cascade, platform evolution, translational hierarchy, and future design priorities, while Table 1, Table 2, Table 3, Table 4, Table 5 and Table 6 summarize material characteristics, drug-delivery strategies, scaffold classes, biological targets, pivotal randomized evidence, and economic considerations.

### Review Scope and Evidence Selection

This article is a focused narrative synthesis rather than a systematic review or meta-analysis. Targeted searches of PubMed-indexed literature were performed through 10 October 2026 using combinations of terms related to coronary stents, drug-coated balloons, bioresorbable scaffolds, vascular healing, endothelial recovery, inflammation, neointimal growth, surface engineering, polymer coatings, drug delivery, and material degradation. Bibliographic details of the cited references were cross-checked against PubMed records before finalizing this draft. Foundational human pathology studies and randomized device trials were considered alongside relevant mechanistic, preclinical, imaging, and contemporary review literature. Evidence was interpreted according to study design: cellular and animal studies were used to support mechanistic hypotheses, human pathology to characterize tissue responses, intravascular imaging to describe structural healing phenotypes, and randomized trials to establish device-specific clinical comparisons. The search was intended to support a focused narrative framework and should not be interpreted as exhaustive systematic evidence retrieval. Generative AI tools (ChatGPT, OpenAI, and Gemini, Google) were used to assist with manuscript organization, language refinement, and conceptual figure development. They were not used to generate or alter research data. All AI-assisted text, references, and graphics were critically reviewed and verified by the authors.

## 2. Biological Basis of Vascular Healing After PCI

### 2.1. Vascular Injury and the Initial Blood–Material Interface

Balloon dilation and stent deployment alter plaque architecture and injure the arterial wall. The extent of endothelial denudation, intimal disruption, and medial damage varies with lesion composition and the intervention. Human coronary pathology has linked strut penetration into the media or lipid core to greater local inflammation [19]. The resulting tissue injury exposes thrombogenic substrates, while the implanted device introduces an additional interface with circulating blood. Early repair therefore begins in a setting in which tissue trauma and foreign-material exposure coexist.

Platelet deposition, thrombus organization, and subsequent cellular ingrowth are part of the early response observed around coronary implants [19]. From an engineering perspective, the initial interface should be considered both a surface and a mechanical problem. A coating may change blood compatibility, but it cannot compensate fully for excessive tissue injury or unfavorable deployment. Conversely, mechanically satisfactory implantation does not eliminate the need for a surface that supports compatible repair. Figure 1 depicts these processes as interacting components rather than a sequence with a single inevitable outcome.

### 2.2. Inflammation and Neointimal Repair

The inflammatory response to implantation contributes to the organization of injured tissue and the development of neointima. Human pathology demonstrates that the severity and location of arterial injury influence subsequent inflammatory reactions [19]. Persistent inflammation can also coexist with delayed healing around drug-eluting devices [2]. The relevant biomaterial objective is therefore to limit sustained injurious responses while permitting the cellular processes required for stable repair.

Neointimal growth provides tissue coverage but can compromise the lumen when excessive. Its inhibition was a major advance of DESs, as demonstrated clinically by RAVEL [1]. However, suppression of neointimal growth should not be equated with comprehensive improvement in healing. An artery with limited tissue growth may still contain uncovered or incompletely healed struts, whereas greater coverage may represent different tissue compositions. These observations support treating restenotic growth, tissue maturation, and thrombogenic vulnerability as related but distinct biological outcomes.

### 2.3. Antiproliferative Drug Effects and Endothelial Function

Local drug delivery must balance inhibition of restenotic tissue growth against the requirements of endothelial repair. Sirolimus-related agents engage mammalian target of rapamycin (mTOR) signaling, but their effects extend beyond the desired inhibition of proliferative responses. In mechanistic experiments, sirolimus–FKBP12.6 interactions altered calcium-dependent signaling, activated protein kinase C-α, and disrupted the p120–vascular endothelial cadherin interaction, impairing endothelial barrier function [20]. This evidence provides a molecular basis for distinguishing endothelial presence from endothelial competence.

The clinical implications of such experiments require careful qualification. Drug concentration, exposure duration, formulation, and the biological model influence the observed response. A pathway demonstrated in cultured cells or experimental tissue does not establish the magnitude of that effect in a contemporary implanted device. Nevertheless, these findings identify endothelial junctional integrity as a relevant design target alongside antiproliferative activity. They also motivate selective or spatially controlled delivery strategies that seek to preserve luminal recovery while controlling tissue growth within the arterial wall.

### 2.4. Structural Coverage and Functional Healing

Optical coherence tomography (OCT) permits detailed assessment of stent apposition, tissue coverage, neointimal thickness, and neoatherosclerotic morphology. Its measurements, however, should be interpreted at the level they directly represent. Experimental fibrin-targeted molecular imaging has shown that apparently covered struts on OCT can contain fibrin-rich tissue rather than mature physiological coverage [21]. Consequently, structural coverage cannot by itself establish a functional endothelial barrier or a nonthrombogenic surface.

For this review, functional endothelial recovery is operationalized as restoration of a continuous barrier with endothelial junctional integrity, antithrombotic behavior, endothelial nitric-oxide synthase (eNOS)/NO activity, low platelet adhesion, and physiologically appropriate vasomotor responsiveness. Favorable healing denotes functional endothelial recovery with controlled inflammation, limited neointimal growth, and restored thromboresistance; maladaptive repair denotes delayed or impaired endothelial recovery accompanied by persistent inflammation, excessive neointimal hyperplasia, neoatherosclerosis, or thrombotic vulnerability. These constructs can be interrogated by complementary methods: histology and scanning electron microscopy for coverage and tissue organization; immunostaining for CD31, VE-cadherin, eNOS, macrophage markers, and fibrin; ex vivo flow or platelet-adhesion assays for thrombogenicity; permeability or junctional assays for barrier function; endothelium-dependent vasomotion or NO-dependent reactivity for physiological recovery; and OCT for apposition, strut coverage, neointimal thickness, and neoatherosclerosis [5,20,21]. No single surrogate endpoint captures all dimensions of vascular healing, and human in vivo assessment of endothelial function remains less direct than structural intravascular imaging.

### 2.5. Neoatherosclerosis and Late Failure

Neoatherosclerosis introduces an additional mechanism of late implant failure beyond conventional neointimal hyperplasia. Human autopsy studies have identified lipid-laden macrophages, necrotic cores, and other atherosclerotic changes within stented neointima, including unstable lesions capable of rupture [22]. In those historical specimens, neoatherosclerosis occurred earlier in DESs than in bare-metal stents (BMS). The findings establish the importance of neointimal tissue quality, although their frequencies should not be extrapolated directly to contemporary devices or unselected living populations.

Late vascular compatibility is thus a longitudinal property. Early tissue coverage does not guarantee durable protection from pathological neointimal transformation. Conversely, successful control of restenosis does not establish restoration of native vascular biology. The molecular evidence of endothelial barrier impairment provides one plausible connection between drug exposure and lipid entry, but clinical neoatherosclerosis remains a multifactorial process [20,22]. Material development should address sustained endothelial function and compatible tissue maturation while recognizing the influence of the underlying atherosclerotic disease.

### 2.6. Translating Biological Processes into Engineering Targets

Taken together, these observations define several complementary objectives: reducing implantation-related injury, limiting early thrombogenicity, controlling excessive neointimal growth, avoiding persistent inflammatory reactions, and promoting functional endothelial recovery. These objectives may conflict when implemented through a single intervention. More potent growth suppression may impede endothelial repair, while reducing the amount of supporting material may alter mechanical performance. We therefore propose coordinated control of the healing environment as the organizing principle for the device comparisons that follow. Figure 1 identifies the intervention points, whereas Figure 4 and Table 4 extend this framework to future multifunctional platforms. The framework is a synthesis of the evidence, not a clinically validated device-selection algorithm.

## 3. Evolution of Coronary Biomaterials

### 3.1. Bare-Metal Platforms and the Importance of Geometry

The metallic coronary stent established a durable intravascular support platform, but tissue responses to its struts became a major determinant of restenosis. The ISAR-STEREO randomized trial provided direct evidence that geometry matters: angiographic restenosis occurred in 15.0% of patients receiving the thinner-strut device compared with 25.8% receiving the thicker-strut device [23]. This comparison supports minimizing unnecessary strut burden, while its specific device designs and historical treatment context limit extrapolation to every current platform.

Strut design should therefore be viewed as part of biomaterial function rather than a purely mechanical specification. Human pathology links injury around individual struts to local inflammatory responses [19], providing a biological rationale for attention to geometry and deployment. The implications extend beyond nominal thickness: the clinically relevant system comprises the platform, its expansion characteristics, and its interaction with the treated lesion.

### 3.2. First-Generation DESs: Local Pharmacology and Delayed Healing

First-generation DESs combined metallic scaffolding with polymer-mediated antiproliferative drug delivery. Their clinical efficacy showed that restenosis could be modified pharmacologically at the device–artery interface [1]. Human pathology subsequently identified incomplete healing and persistent fibrin around some implanted devices, with uncovered struts contributing to late thrombotic risk [2]. These findings revealed a design trade-off between suppressing tissue growth and permitting compatible vascular repair.

The historical experience should be interpreted at the level of the complete device. Drug, polymer, strut architecture, implantation conditions, and patient characteristics were not independent variables in most clinical comparisons. It would therefore be inappropriate to attribute every adverse response to a single component or to generalize first-generation pathology to all DESs. The enduring lesson is that antirestenotic efficacy requires assessment together with healing and safety.

### 3.3. Contemporary DESs and Polymer Strategies

Later-generation DESs incorporated changes in platform design, drug delivery, and polymer compatibility. Human comparisons of second-generation everolimus-eluting stents with first-generation devices documented more favorable pathological healing [3]. Durable polymers can remain part of an effective device system, whereas biodegradable polymers seek to reduce persistent coating exposure after drug release. These are alternative engineering strategies whose clinical performance depends on the entire platform.

The BIOSCIENCE trial illustrates why polymer labels cannot serve as a hierarchy of clinical quality. At five years, target lesion failure was similar with an ultrathin-strut biodegradable-polymer sirolimus-eluting stent and a thin-strut durable-polymer everolimus-eluting stent: 20.2% versus 18.8%, respectively. Definite stent thrombosis was 1.6% in both groups [24]. The comparison simultaneously involved differences in strut design, drug, and polymer; it cannot isolate the effect of polymer degradation. Biodegradation is consequently a material property to characterize, rather than sufficient evidence of clinical superiority.

### 3.4. Polymer-Free Drug Delivery

Polymer-free platforms remove the conventional polymer carrier while retaining local drug delivery and permanent metallic support. In LEADERS FREE, a polymer-free umirolimus-coated stent was superior to BMSs for the trial’s safety and efficacy endpoints in patients at high bleeding risk treated with one month of dual antiplatelet therapy [25]. This established the efficacy of a particular drug-coated platform under a tested antiplatelet regimen.

The comparator is essential to the interpretation. Superiority over BMSs does not demonstrate superiority over contemporary polymer-based DESs, nor does it prove that polymer absence caused the observed benefit. Polymer-free systems also retain requirements for reproducible drug deposition, release, and tissue exposure. Table 2 therefore compares drug-delivery characteristics across device systems rather than classifying polymer presence as intrinsically favorable or unfavorable.

### 3.5. DCBs: Transient Delivery Without a New Permanent Scaffold

DCBs separate local drug administration from the routine implantation of another permanent scaffold, but the treatment strategy is inseparable from drug formulation, carrier technology, lesion preparation, and the threshold for bailout or rescue stenting. The AGENT IDE trial demonstrated clinical benefit of a paclitaxel-coated balloon over uncoated balloon angioplasty for in-stent restenosis [6]. This indication is biologically and mechanically distinct from de novo disease because treatment avoids an additional stent layer while acting within an already scaffolded segment.

In de novo disease, trial results vary with patient selection, vessel size, balloon technology, procedural success criteria, and rescue-stenting strategy. BASKET-SMALL 2 showed noninferiority of a DCB strategy to second-generation DESs in small-vessel disease after successful lesion preparation [26], whereas REC-CAGEFREE I, which enrolled a broader de novo population and permitted protocol-guided rescue stenting, did not establish noninferiority of the tested DCB strategy and showed a higher device-oriented event rate [7]. A 2026 prespecified analysis reported higher event risk with DCB treatment in both long and short lesion groups without a significant interaction by lesion length [27]. These findings should not be interpreted as a uniform DCB class effect: drug identity, excipient/carrier, coating transfer, lesion preparation, dissection/recoil tolerance, and rescue-stenting criteria are components of the tested treatment strategy.

### 3.6. Bioresorbable Scaffolds: Matching Support to Resorption

Bioresorbable scaffolds seek to provide initial support followed by reduction in permanent foreign-material burden. Their evaluation requires attention to the relationship between mechanical support, tissue repair, and material degradation. The ABSORB IV trial showed that, despite improved implantation technique, five-year target lesion failure was higher with the studied polymeric scaffold than with cobalt-chromium everolimus-eluting stents: 17.5% versus 14.5% [28]. Event rates were similar between years three and five, but this later convergence did not reverse the accumulated difference.

Longer follow-up has also challenged the assumption of an eventual late advantage. In COMPARE-ABSORB, the three-to-seven-year landmark comparison did not demonstrate superiority of the scaffold, and clinically indicated target lesion revascularization was higher in the scaffold group [8]. These results concern the investigated polymeric device and should not be treated as definitive evidence about every resorbable material. Early BIOMAG-I findings with a newer magnesium scaffold were encouraging, but its first-in-human, nonrandomized design cannot establish comparative effectiveness against contemporary DESs [29]. Table 3 provides the material-specific framework for the detailed scaffold discussion.

### 3.7. Bioactive Surfaces and Multifunctional Platforms

Bioactive surface engineering seeks to influence blood and cellular responses directly. The CD31-mimetic coating study provides a preclinical example of combined promotion of endothelialization and attenuation of inflammation and neointimal growth [5]. Its translational appeal lies in addressing several aspects of healing together. However, experimental biological performance does not establish durability during clinical delivery, long-term safety, or benefit in patient outcomes.

Figure 2 and Table 1 should therefore be interpreted as an evolution of design objectives with overlapping technological branches. Biodegradable-polymer DESs, polymer-free platforms, DCBs, bioresorbable scaffolds, and bioactive surfaces are not a universal chronological sequence or a ladder of proven superiority. Their shared challenge is to coordinate mechanical performance with local biological control. This perspective sets the basis for Section 4, which examines DESs as integrated platform–coating–drug systems, followed by the device-specific and translational assessments in Section 5, Section 6, Section 7, Section 8 and Section 9.

**Table 1:** Evolution of biomaterial strategies in coronary intervention.

Platform	Principal Material/Components	Primary Objective	Major Strengths	Principal Limitations
Bare-metal stent (BMS)	Stainless steel, cobalt–chromium, or platinum–chromium scaffold	Permanent mechanical scaffolding and acute lumen gain	Reliable radial support; predictable deployment	Neointimal hyperplasia and restenosis [23]
Durable-polymer DES	Metallic scaffold + permanent polymer + antiproliferative drug	Sustained local inhibition of VSMC proliferation	Controlled drug release; strong antirestenotic efficacy	Persistent polymer/metal exposure; potential delayed healing [2,3,9,10]
Biodegradable-polymer DES	Metallic scaffold + degradable polymer + drug	Temporary polymer-mediated drug delivery	Reduced long-term polymer burden	Outcome depends on complete platform and degradation kinetics [9,10,24]
Polymer-free DES	Metallic scaffold with surface reservoir or drug coating	Drug delivery without a permanent polymer	Eliminates durable-polymer exposure	Drug-release control remains device-specific; no proven class superiority [25,30]
Drug-coated balloon (DCB)	Balloon + antiproliferative drug + carrier/excipient	Rapid local drug transfer without new permanent implantation	Leave-nothing-behind strategy	No sustained mechanical support; lesion preparation and transfer efficiency are critical [6,7,11,26,31,32]
Bioresorbable scaffold (BRS)	Polymeric or bioresorbable metallic scaffold + drug/coating	Temporary scaffolding followed by resorption	Potential reduction in long-term foreign-material burden	Radial strength, strut thickness, degradation kinetics, and thrombogenicity [8,12,13,28,29]
Bioactive/biomimetic platform	Functionalized surface incorporating biological or molecular components	Direct modulation of thrombosis, inflammation, and endothelial healing	Potential multifunctional, healing-directed therapy	Manufacturing complexity and limited clinical validation [5,14,15,16,17]

## 4. Drug-Eluting Stents: Engineering the Drug–Polymer–Vessel Interface

### 4.1. The DES as an Integrated Biomaterial System

A contemporary DES should be considered an integrated system rather than the sum of an isolated metal, polymer, and drug. The platform determines radial support, conformability, deliverability, strut geometry, and the magnitude of local flow disturbance. The coating governs drug loading, release kinetics, interfacial chemistry, and—when biodegradable—degradation behavior. The drug modifies proliferative and inflammatory pathways but may also affect endothelial recovery. These components interact mechanically and biologically, which explains why apparently similar DES classes can differ in healing despite sharing the same nominal drug or broad polymer category [9,10].

This systems perspective is important when interpreting comparative studies. An ultrathin biodegradable-polymer stent differs from a thin-strut durable-polymer stent in more than one attribute, and clinical noninferiority or superiority cannot be assigned to polymer behavior alone. The BIOSCIENCE findings discussed above illustrate this principle [24]. For material science, the clinically meaningful unit is therefore the complete implanted platform, including its geometry, coating integrity, elution profile, and deployment behavior.

### 4.2. Metallic Platforms, Strut Geometry, and Local Hemodynamics

Modern coronary stents predominantly use cobalt–chromium or platinum–chromium alloys to achieve sufficient radial strength with thinner struts than earlier stainless-steel platforms. Reducing strut thickness can lessen vessel-wall injury and protrusion into the lumen while improving deliverability; historical randomized data support a relationship between strut thickness and restenosis [23]. However, thinner is not an unlimited design objective. Adequate radial strength, radiopacity, fracture resistance, and expansion capacity remain necessary, particularly in calcified, ostial, bifurcation, and long lesions.

Strut geometry also changes local flow. Computational and experimental studies show that protruding struts generate recirculation, low-shear regions, and prolonged particle residence, while thicker or malapposed struts increase thrombogenicity. In matched ex vivo and porcine coronary experiments, thick-strut devices produced greater thrombus burden than otherwise similar thin-strut platforms, and modeled flow disturbances colocalized with clot formation [33]. Thus, the biological effect of a strut reflects not only material exposure but also the hemodynamic microenvironment created by strut height, shape, spacing, and apposition. The design objective is therefore to minimize flow-disruptive material while preserving adequate radial strength, expansion capacity, fracture resistance, and deliverability.

### 4.3. Polymer Chemistry as a Biological Interface

Polymers in DESs were initially viewed mainly as reservoirs that enabled controlled drug release. Current evidence supports a broader interpretation. Surface chemistry, wettability, charge, molecular mobility, degradation products, and coating integrity influence protein adsorption, coagulation, complement activation, leukocyte responses, and endothelial recovery [9]. Durable polymers may provide highly reproducible release profiles and stable coatings, whereas biodegradable polymers are designed to disappear after drug delivery. Neither category is biologically uniform, and the behavior of a specific polymer cannot be inferred solely from whether it is permanent or degradable.

Biodegradation also introduces molecular and interfacial consequences. PLLA and related aliphatic polyesters undergo hydrolytic ester-bond cleavage, producing progressively lower-molecular-weight oligomers and ultimately lactic-acid-derived products. When generation exceeds local diffusion and buffering, the peripolymer microenvironment can become more acidic, which may alter protein adsorption/conformation and amplify inflammatory cell activation. Experimental exposure to low-molecular-weight polyester particles has induced macrophage and endothelial cytotoxicity, increased inflammatory signaling, reduced endothelial NO production, and accelerated further polymer degradation through protease release [34]. These findings support a feedback model in which degradation chemistry can influence macrophage activation and endothelial dysfunction; however, direct evidence linking a specific coronary scaffold to a defined macrophage-polarization state remains limited. The in vivo effect depends on polymer mass, crystallinity, degradation rate, tissue clearance, and local buffering, so clinically meaningful acidity should not be assumed from hydrolysis alone.

### 4.4. Drug Identity, Dose, and Release Kinetics

Paclitaxel and limus-family agents suppress neointimal growth through different molecular mechanisms. Paclitaxel stabilizes microtubules and can inhibit migration and induce apoptosis, whereas sirolimus-family agents primarily produce cytostatic mTOR-mediated cell-cycle arrest. Direct cellular comparisons have demonstrated drug-, dose-, exposure-time-, and cell-type-dependent effects on coronary smooth muscle and endothelial cells [35]. Contemporary DESs predominantly use sirolimus, everolimus, zotarolimus, or biolimus derivatives because their pharmacology can be coupled to controlled release from thin polymer coatings, but the biological effect of any agent remains dependent on local dose, tissue concentration, release duration, and spatial distribution.

This balance explains why release kinetics are a central material property. Excessive early drug loss may compromise antirestenotic efficacy, whereas prolonged high local exposure may delay recovery. The design target is not maximum drug retention but sufficient and appropriately timed tissue exposure. Newer delivery concepts increasingly treat pharmacokinetics as an adjustable feature of the device rather than a fixed consequence of the selected drug [9,10].

### 4.5. Polymer-Free Approaches

Polymer-free DESs seek to eliminate the conventional polymer carrier by using microporous, nanoporous, grooved, or reservoir-based metallic surfaces for drug loading and release. The conceptual advantage is straightforward: one persistent foreign material is removed. However, polymer absence does not remove the metallic scaffold and does not guarantee superior healing. It also makes control of drug loading and elution a surface-engineering problem.

Clinical interpretation must therefore remain device-specific. LEADERS FREE established benefit of one polymer-free drug-coated stent relative to a BMS in high-bleeding-risk patients [25], but this should not be generalized into class superiority over contemporary polymer-based DESs. Indeed, in ONYX ONE, a polymer-based zotarolimus-eluting stent was noninferior to a polymer-free umirolimus-coated stent for both the prespecified safety composite and target-lesion failure at 1 year in patients at high bleeding risk treated with 1 month of dual antiplatelet therapy [30]. Polymer-free technology is therefore best regarded as one strategy for optimizing the material–drug interface rather than an endpoint in itself.

### 4.6. Clinical Translation of DES Material Engineering

Contemporary DES performance reflects the accumulated effects of thinner struts, more biocompatible surfaces, improved elution, and better implantation technique. Yet late target-lesion events continue to occur [4], indicating that permanent implantation still creates a long-term vessel–device relationship. This residual hazard does not invalidate DESs; rather, it defines the benchmark against which alternative technologies must be judged.

The practical lesson is that material engineering should be evaluated hierarchically. First, a platform must maintain mechanical integrity and deliver its drug reproducibly. Second, it should support compatible healing with limited thrombogenicity and inflammation. Third, it must demonstrate durable clinical safety and efficacy in the indication for which it is intended. Table 2 contrasts this scaffold-based paradigm with the transient drug-delivery strategy of DCBs.

**Table 2:** Comparison of drug-eluting stents and drug-coated balloons as local drug-delivery platforms.

Feature	Drug-Eluting Stent (DES)	Drug-Coated Balloon (DCB)	Biological/Clinical Implication
Permanent implant	Yes	No	DES maintains chronic vessel–material interaction; DCB avoids a new permanent implant [11]
Mechanical support	Sustained scaffolding	None after balloon withdrawal	DES can prevent recoil; DCB requires satisfactory lesion preparation [11,26]
Drug-delivery mechanism	Controlled release from coating/reservoir	Rapid transfer during balloon inflation followed by tissue retention	Distinct pharmacokinetic profiles influence efficacy and healing [9,10,11,31,32]
Predominant drugs	Mainly limus-family agents	Paclitaxel or sirolimus formulations	Drug physicochemical properties and carrier technologies determine uptake and retention [10,11,31,32]
Polymer/carrier system	Durable polymer, biodegradable polymer, or polymer-free	Excipients, carriers, nanoparticles, or other transfer matrices	Delivery matrices influence release, transfer, and biocompatibility [9,11,31,32]
Duration of material exposure	Long-term metallic scaffold; polymer exposure varies	Transient balloon contact only	DCB minimizes chronic device-related vessel-wall interaction [11]
Endothelial healing	Influenced by strut, polymer, drug dose, and kinetics	No retained scaffold after drug transfer	Absence of a new scaffold may facilitate restoration, but clinical benefit is indication-specific [6,7,26]
Main limitation	Persistent implant-related late events may occur	Recoil/dissection, geographic miss, or inadequate drug transfer	Failure mechanisms differ between scaffold-based and transient strategies [4,7,11]
Therapeutic concept	Scaffold + local drug delivery	Transient local drug delivery	Two distinct approaches to balancing mechanics and vascular healing [11]

## 5. Drug-Coated Balloons as Transient Drug-Delivery Platforms

### 5.1. Separating Drug Delivery from Permanent Scaffolding

DCBs represent a fundamentally different biomaterial concept from DESs. During a short balloon inflation, an antiproliferative agent is transferred from the balloon surface to the arterial wall; the delivery device is then removed. The vessel is therefore exposed to a transient drug–carrier interface rather than a newly implanted permanent scaffold. This approach is attractive where adequate lumen gain can be achieved without leaving additional metal, particularly in ISR and selected de novo lesions [11].

The absence of a permanent implant should not be interpreted as absence of material engineering. DCB performance depends heavily on the formulation placed on the balloon surface, including drug crystallinity, dose, excipient or carrier, coating morphology, adhesion during delivery, transfer efficiency at inflation, and tissue retention. In this sense, a DCB is a short-contact biomaterial platform whose success depends on delivering an adequate pharmacological payload while minimizing upstream loss.

### 5.2. Paclitaxel: Physicochemical Suitability for Rapid Transfer

Paclitaxel became the dominant early DCB drug because of its high lipophilicity, rapid tissue uptake, and prolonged intracellular retention. These properties are advantageous when drug transfer must occur within seconds rather than over weeks. Excipients such as iopromide, urea, or other carrier systems help control coating stability and facilitate release from the balloon surface to tissue [11].

However, the pharmacology that makes paclitaxel suitable for a balloon differs from the logic of contemporary DESs. High local transfer and persistence are desired, but particle loss before lesion contact and heterogeneous deposition can reduce efficiency. Device performance therefore depends on the combination of drug, excipient, coating process, balloon handling, and lesion preparation rather than on paclitaxel alone.

### 5.3. Sirolimus-Coated Balloons and Carrier Engineering

Sirolimus and its analogues are attractive because of their cytostatic mechanism and extensive DES experience, but lower lipophilicity creates a delivery challenge when exposure time is brief. Sirolimus-coated balloon platforms have therefore relied on carrier technologies such as phospholipid encapsulation, nanoparticles, micro-reservoirs, or crystalline formulations to enhance arterial uptake and retention.

Comparative evidence remains formulation-dependent. Meta-analyses suggest broadly similar short-term clinical outcomes between paclitaxel- and sirolimus-coated balloons, with some angiographic measures favoring paclitaxel [31,32]. Randomized head-to-head data in de novo lesions have shown noninferiority of one crystalline sirolimus-coated balloon to a clinically established paclitaxel-coated balloon for 6-month late lumen loss, but the study was small and used specific formulations after mandatory successful lesion preparation [36]. These findings argue against treating all sirolimus-coated balloons—or all DCBs—as interchangeable classes. Drug crystallinity, excipient or carrier, coating stability, transfer efficiency, and tissue retention are integral device attributes.

### 5.4. Lesion Preparation as Part of the Biomaterial Strategy

A DCB has no capacity to maintain lumen dimensions after withdrawal. Successful therapy therefore depends on adequate lesion preparation, acceptable residual stenosis, preserved flow, and predefined management of recoil or flow-limiting dissection. Trials have differed in the use of scoring/cutting balloons, acceptable residual stenosis, dissection thresholds, vessel size, and bailout stenting. These procedural variables alter both the population that ultimately receives DCB-only therapy and the mechanical substrate on which drug transfer occurs; lesion preparation should therefore be regarded as part of the biomaterial treatment strategy rather than a background procedural detail.

This consideration helps explain divergent trial results. AGENT IDE evaluated a paclitaxel-coated balloon in ISR against uncoated balloon angioplasty [6]. BASKET-SMALL 2 randomized patients with small de novo vessels only after successful predilatation and found 12-month noninferiority to DESs [26]. REC-CAGEFREE I enrolled non-complex de novo lesions without a vessel-diameter restriction after successful predilatation, used a paclitaxel-coated balloon with protocol-guided rescue stenting, and reported a 9.4% rescue-stent rate; the DCB strategy failed noninferiority for the 24-month device-oriented composite endpoint and remained less favorable at longer follow-up [7]. Accordingly, outcomes should be interpreted according to patient population, lesion phenotype, DCB formulation, lesion-preparation protocol, and rescue-stenting strategy rather than as a universal comparison of ‘DCB versus DES’.

### 5.5. Healing Implications and Remaining Questions

Removing a new permanent scaffold eliminates chronic exposure to additional metal, polymer, and struts and may preserve vasomotion or adaptive remodeling in successfully treated segments. These are plausible biological advantages but should not be assumed to translate automatically into fewer late events. Recoil, residual dissection, inadequate drug transfer, geographic miss, or heterogeneous tissue retention can offset the theoretical benefits.

Future DCB development is therefore likely to focus on more reproducible coating transfer, lower drug loss during navigation, improved tissue retention, and drug-carrier combinations tailored to lesion biology. Smart or stimuli-responsive balloon technologies remain investigational but illustrate how transient delivery could evolve beyond a single bolus exposure. These concepts are revisited in Section 9.

## 6. Bioresorbable Scaffolds and Temporary Vascular Support

### 6.1. The Rationale for Resorbable Scaffolding

Bioresorbable scaffolds attempt to reconcile two competing objectives: the need for temporary radial support after PCI and the desire to avoid lifelong vessel caging. In principle, an ideal scaffold would provide DES-like early mechanical performance and drug delivery, maintain support through the period of recoil and vessel healing, and then disappear without leaving harmful degradation products or a prolonged inflammatory response.

First-generation polymeric scaffolds demonstrated the difficulty of achieving this balance. Thick struts, limited radial strength, complex implantation requirements, and prolonged resorption contributed to adverse early and intermediate outcomes [28]. The important lesson is not that resorption is undesirable, but that the kinetics of mechanical loss and material disappearance must be synchronized with vascular repair.

### 6.2. Polymeric Scaffolds

Poly-L-lactic acid (PLLA) became the archetypal scaffold material because it can be processed into a temporary structure and undergo hydrolytic degradation. Its relatively low elastic modulus, however, requires greater strut dimensions to achieve radial strength comparable with metal. Thick struts increase crossing profile, disturb flow, and increase the surface area exposed to blood. In addition, degradation is prolonged, so the theoretical benefit of complete disappearance is delayed.

ABSORB IV and COMPARE-ABSORB showed that improved implantation technique did not eliminate the clinical limitations of the investigated first-generation PLLA-based scaffold [8,28]. These findings should be restricted to that polymeric platform and its implantation era, rather than extrapolated to all resorbable scaffolds. The broader lesson is that any bioresorbable system must demonstrate acceptable outcomes during the entire interval in which it retains mechanical and biological relevance, not merely eventual material disappearance.

### 6.3. Magnesium-Based Scaffolds

Magnesium alloys offer a different balance between mechanics and degradation. In aqueous physiological environments, Mg corrosion releases Mg^2+^ while generating hydroxide and hydrogen; Mg(OH)_2_ and other corrosion products form at the surface, and chloride-rich conditions can destabilize protective layers and sustain corrosion. The resulting microenvironment may therefore include local alkalinization, hydrogen evolution, elevated Mg^2+^ concentrations, and deposition of corrosion products. Experimental vascular studies show that excessive Mg release and pH shifts can impair endothelial and smooth-muscle-cell viability and endothelial-dependent vasorelaxation, whereas corrosion-controlling surface treatments mitigate these effects [37]. Thus, degradation rate is simultaneously a mechanical and biological design variable.

The challenge is that degradation cannot occur too quickly. Premature loss of radial strength may permit recoil before tissue stabilization, whereas excessive local corrosion may disrupt cellular function despite the physiological role of Mg. BIOMAG-I provided encouraging first-in-human feasibility data for the DREAMS 3 magnesium scaffold [29], but this constitutes early clinical feasibility rather than comparative effectiveness against contemporary DESs. Magnesium should therefore be described as clinically advanced among bioresorbable metals while still requiring adequately powered randomized comparisons.

### 6.4. Iron- and Zinc-Based Materials

Iron provides high mechanical strength and enables thin-strut scaffold designs, but its relatively slow corrosion can prolong material persistence. Long-term porcine studies have supported the feasibility of controlled iron corrosion and vascular biocompatibility. More recently, the IRONMAN II randomized trial advanced iron scaffolds beyond purely preclinical development by comparing a thin-strut sirolimus-eluting iron bioresorbable scaffold with cobalt–chromium everolimus-eluting stents [38]. This emerging evidence should still be interpreted as device-specific rather than proof of a class effect for iron-based scaffolds.

Zinc remains less clinically mature. Its intermediate corrosion profile is attractive because it may avoid both the rapid mechanical loss of inadequately controlled Mg systems and the prolonged persistence of slowly degrading iron. Primary studies in porcine coronary models have reported endothelialization, maintained patency, gradual degradation, and acceptable local tissue responses with selected zinc alloys, including Zn-Cu-Mn platforms [39]. However, zinc coronary scaffolds remain predominantly preclinical, and comparative human evidence is sparse. Together, the Fe and Zn literature illustrates that alloy chemistry can be used as a direct control variable for radial strength, corrosion kinetics, ion release, and vascular response.

### 6.5. Resorption as a Time-Dependent Design Variable

The central engineering parameter for a BRS is not whether it disappears, but when and how. Three coupled trajectories must be aligned: loss of radial strength, tissue stabilization, and material mass loss. If mechanical support is lost before the vessel is stable, recoil becomes possible. If mass persists long after support is no longer necessary, the theoretical reduction in chronic foreign-body burden is delayed. If degradation products trigger inflammation, resorption may introduce a new source of biological injury.

Accordingly, future BRS development should be assessed using time-resolved mechanical, imaging, biological, and clinical endpoints. Table 3 summarizes the principal material classes and their characteristic trade-offs. The BRS field is increasingly shifting from the binary concept of “resorbable versus permanent” toward control of degradation kinetics, strut architecture, and surface biofunction [12,13].

**Table 3:** Bioresorbable scaffold materials and their biological characteristics.

Material Class	Key Characteristics	Potential Advantages	Major Challenges	Expected Vascular Implications
PLLA-based polymeric scaffold	Hydrolytically degradable polymer; relatively low elastic modulus	Complete eventual resorption	Thick struts, limited radial strength, prolonged resorption	Potential restoration after resorption, but early flow/healing penalties may occur [12,28]
Magnesium-based scaffold	Bioresorbable metal with relatively rapid degradation	Metal-like early support with eventual disappearance	Premature loss of strength if degradation is excessive	Promising temporary support; randomized comparative evidence remains limited [12,13,29]
Iron-based scaffold	High mechanical strength; relatively slow corrosion	Strong radial support; potential thin-strut design	Slow degradation and persistent residue	May prolong foreign-material exposure despite excellent mechanics [13]
Zinc-based scaffold	Intermediate corrosion profile	Potential balance between support and degradation	Limited clinical experience	Conceptually attractive but not yet clinically mature [13]
Hybrid/composite platform	Combination of polymers, metals, coatings, drugs, or surface modification	Simultaneous optimization of mechanics, degradation, and healing	Manufacturing and regulatory complexity	Potential for more individualized control of the scaffold–vessel interaction [12,13]

## 7. Bioactive and Biomimetic Surface Engineering

### 7.1. From Passive Compatibility to Active Biological Modulation

Conventional device design seeks to minimize harmful interactions with blood and tissue. Bioactive surface engineering goes further by attempting to direct specific biological responses. Surface roughness, charge, wettability, chemical functionality, and molecular mobility influence the composition and conformation of adsorbed proteins, which in turn affect platelet adhesion, coagulation, complement activation, leukocyte recruitment, and endothelial attachment [9,14]. The device surface is therefore an active determinant of the initial biological environment.

Surface engineering can be broadly divided into passive and active strategies. Passive approaches seek to reduce nonspecific protein and platelet adhesion by creating inert or highly hydrated interfaces. Active approaches immobilize or release molecules that directly influence coagulation, inflammation, endothelialization, or tissue growth. In practice, next-generation coatings increasingly combine both concepts.

### 7.2. Hemocompatible and Antithrombotic Surfaces

Hemocompatibility can be improved by hydrophilic or zwitterionic interfaces, anticoagulant coatings, heparin-like chemistries, and systems that generate or release nitric oxide (NO) [14,15]. Primary studies support biological effects beyond simple surface coverage. For example, a mechanically tough NO-eluting hydrogel coating reduced platelet-related thrombogenicity, promoted endothelial-cell adhesion, inhibited smooth-muscle-cell responses, and reduced inflammation and neointimal hyperplasia in rabbit and swine vascular models [40]. Such studies provide functional antithrombotic and cellular endpoints rather than relying solely on morphological coverage.

The translational challenge is durability after real device handling. A coating that performs well on a flat coupon may crack, delaminate, or lose activity during sterilization, crimping, catheter tracking, balloon expansion, or prolonged blood exposure. Therefore, surface studies should report coating integrity after deformation together with platelet/coagulation assays and biological function. Recent materials studies have explicitly tested deformation adaptability during stent crimping and expansion, illustrating the type of mechanical validation required before biological performance can be considered device-relevant [41].

### 7.3. Pro-Endothelial and Biomimetic Interfaces

Rapid endothelial coverage is often presented as a desirable endpoint, but functional endothelialization is more important than coverage alone. A newly formed layer should provide barrier integrity, antithrombotic behavior, eNOS/NO signaling, and physiological responsiveness to shear. Primary biomimetic studies support this distinction: CD31-mimetic coatings reduced platelet/leukocyte activation and promoted a continuous endothelial layer in porcine coronary arteries [5], while VE-cadherin-targeted zwitterionic stents reduced platelet adhesion and promoted endothelial healing in rabbit vascular models [42]. These endpoints extend beyond the presence of a cell layer and begin to test whether the regenerated interface behaves more like native endothelium.

Pro-endothelial strategies include immobilized peptides or adhesion ligands, endothelial progenitor-cell capture, extracellular-matrix-mimetic coatings, CD31-related approaches, and surfaces that favor endothelial cells over VSMCs. The CD31-mimetic coating discussed in Section 1 provides a representative preclinical example [5]. The broader design principle is selectivity: a successful surface should encourage endothelial recovery without simultaneously promoting excessive neointimal proliferation.

### 7.4. Anti-Inflammatory and Immunomodulatory Surface Design

Macrophage and leukocyte responses are central to foreign-body reactions. Surface chemistry, adsorbed-protein conformation, degradation products, and released ions can alter macrophage activation and cytokine signaling. Polyester degradation products have produced macrophage cytotoxicity and inflammatory activation in vitro [34], while emerging multifunctional coatings increasingly seek to shift macrophage responses toward a less inflammatory phenotype. Direct evidence that a specific coronary coating produces durable, functionally beneficial macrophage polarization after deployment remains limited, however, and this should be considered an investigational rather than established mechanism.

This field is increasingly converging with biomimetic engineering. Rather than simply eluting a single anti-inflammatory agent, surfaces can be designed to resemble aspects of extracellular matrix or endothelial glycocalyx, thereby changing how cells and plasma proteins interact with the device. Recent reviews describe rapid growth in functional coatings intended simultaneously to inhibit intimal hyperplasia and promote re-endothelialization [17].

### 7.5. Nanostructured and Multifunctional Coatings

Nanoporous reservoirs, nanoparticles, nanofibrous interfaces, and nanoscale patterning can regulate both drug delivery and cellular behavior. Their potential advantage is multifunctionality: one architecture may combine antithrombotic activity, endothelial selectivity, drug release, and immunomodulation. Primary coating studies increasingly test multiple functional endpoints, but evidence of post-crimping/expansion integrity, long-term activity, and reproducible manufacturing remains inconsistent [40,41,42]. Thus, multifunctionality should be judged not by the number of incorporated components but by retained function after device processing and deployment.

The central translational question is therefore whether biological sophistication yields robust benefit under the mechanical stresses of real PCI. Table 4 summarizes the biological targets and candidate engineering approaches. Figure 4 integrates these strategies into a future multifunctional platform.

**Table 4:** Biological targets and engineering strategies for next-generation coronary biomaterials.

Biological Target	Desired Response	Candidate Engineering Strategies	Potential Translational Benefit
Platelet adhesion/activation	Reduce thrombogenicity	Hemocompatible coatings; NO-releasing or antithrombotic surfaces	Potential reduction in device-related thrombosis [28,29]
Endothelial recovery	Rapid functional endothelial coverage	Pro-endothelial coatings; cell-capturing or biomimetic surfaces	Restoration of a thromboresistant luminal interface [5,30,38]
VSMC proliferation	Control excessive neointimal growth without excessive healing delay	Optimized local antiproliferative drug delivery	Reduced restenosis with preserved repair [1,19,20]
Inflammatory response	Reduce chronic inflammation and foreign-body reaction	Biocompatible/degradable coatings; anti-inflammatory surface design	Improved long-term vessel compatibility [2,19,28,38]
Drug transfer/retention	Efficient local tissue exposure with minimal systemic loss	Nanocarriers; porous reservoirs; optimized DCB excipients	Improved local efficacy and dose efficiency [22,23,24,33]
Material persistence	Temporary support followed by controlled disappearance	Bioresorbable polymers and Mg/Fe/Zn alloys	Reduced long-term foreign-material burden [26,27]
Oxidative/inflammatory microenvironment	Adaptive treatment according to local signals	ROS-, pH-, enzyme-, or inflammation-responsive systems	Potential on-demand local therapy; predominantly investigational [31,32]
Multifunctional healing	Coordinate antithrombotic, anti-inflammatory, and pro-endothelial effects	Multilayer coatings; biomimetic interfaces; multifunctional surfaces	Integrated control of competing healing pathways [28,29,30,38]
Patient/lesion heterogeneity	Match material/device to vascular environment	Imaging-guided selection and phenotype-based treatment	Potential precision PCI; currently a conceptual framework rather than a validated algorithm

**Table 5:** Selected pivotal randomized comparisons linking coronary biomaterial strategies to clinical outcomes.

Trial	Population/Indication	Device/Comparator	Endpoint/Follow-Up	Key Result/Interpretation
RAVEL [1]	De novo coronary lesions	Sirolimus-eluting stent vs. BMS	Angiographic restenosis/late loss; 6 months	Marked suppression of neointimal restenosis; established local antiproliferative efficacy.
BIOSCIENCE [24]	All-comers PCI	Biodegradable-polymer sirolimus DES vs. durable-polymer everolimus DES	Target lesion failure; 5 years	Similar TLF; polymer strategy cannot be isolated from total device design.
LEADERS FREE [25]	High bleeding risk	Polymer-free biolimus-A9 DCS vs. BMS	Safety composite and clinically driven TLR; 1 year	Improved safety/efficacy versus BMS with short DAPT; comparator limits class inference.
ONYX ONE [30]	High bleeding risk	Durable-polymer zotarolimus DES vs. polymer-free biolimus-A9 stent	Cardiac death/MI/ST and TLF; 1 year	Polymer-based DES was noninferior; polymer elimination did not confer class-wide superiority.
BASKET-SMALL 2 [26]	Small-vessel de novo CAD	Paclitaxel DCB vs. second-generation DES	MACE; 12 months	DCB was noninferior after successful lesion preparation; strategy- and indication-specific.
AGENT IDE [6]	Coronary ISR	Paclitaxel-coated balloon vs. uncoated balloon	Target lesion failure; 1 year	DCB reduced TLF versus uncoated balloon in ISR.
REC-CAGEFREE I [7]	Non-complex de novo CAD	Paclitaxel DCB + protocol rescue stenting vs. planned sirolimus DES	Device-oriented composite endpoint; 24 months/3 years	DCB strategy did not meet noninferiority; device and procedural context were critical.
ABSORB IV [28]	De novo CAD	PLLA everolimus BVS vs. cobalt–chromium EES	Target lesion failure; 1 and 5 years	Optimized implantation did not establish long-term superiority; applies to the investigated PLLA scaffold.
COMPARE-ABSORB [8]	Higher restenosis-risk PCI	PLLA everolimus BVS vs. metallic EES	Target lesion failure; 7 years	No late post-resorption superiority; device-specific limitations persisted.
IRONMAN II [38]	De novo CAD	Sirolimus-eluting iron BRS vs. cobalt–chromium EES	2-year in-segment late lumen loss	Noninferior to cobalt–chromium EES for 2-year in-segment late lumen loss; QFR and OCT flow area were also noninferior, while longer-term benefit after complete resorption remains to be established.

## 8. From Biomaterial Properties to Imaging Phenotypes and Clinical Outcomes

### 8.1. A Translational Continuum

Figure 3 summarizes the central translational model of this review: material properties shape cellular responses; cellular responses produce tissue-level healing phenotypes; these phenotypes can be sampled by intravascular imaging; and clinical events emerge from the integrated behavior of the vessel, device, procedure, and patient. The model is intentionally directional but not deterministic. Each arrow represents a plausible and partly measurable relationship rather than proof of mediation.

This distinction is important because biomaterial studies often stop at an intermediate endpoint. Reduced platelet adhesion in vitro, greater endothelial coverage in animals, or more complete OCT strut coverage are useful signals, but none alone establishes clinical benefit. Translation requires concordance across progressively higher levels of evidence.

### 8.2. OCT as a Structural Healing Tool

OCT provides near-histological axial resolution and can assess strut apposition, tissue coverage, neointimal thickness, and features of neoatherosclerosis. These measurements are particularly useful for comparing device healing because they can be obtained repeatedly in living patients. OCT can identify uncovered or malapposed struts and characterize the spatial heterogeneity of tissue response.

However, OCT measures optical morphology rather than endothelial function. As noted in Section 2, fibrin-rich tissue may appear as coverage [21]. Likewise, an apparently homogeneous neointima does not establish normal endothelial barrier function, vasomotor behavior, or absence of prothrombotic signaling. Imaging therefore occupies an intermediate position between mechanism and clinical outcome.

### 8.3. Neoatherosclerosis and Late Device Phenotypes

Neoatherosclerosis illustrates why late imaging and pathology remain important. Lipid accumulation, macrophage infiltration, calcification, and neointimal plaque rupture can develop within previously treated segments [22]. Contemporary devices may differ from historical first-generation DESs in the timing and frequency of these processes, but the concept remains relevant: long-term success requires durable tissue compatibility, not merely early suppression of restenosis.

Material characteristics may contribute indirectly through endothelial dysfunction, inflammation, altered flow, or prolonged foreign-body exposure. Yet patient-level atherosclerotic biology remains a major determinant. Neoatherosclerosis should therefore be interpreted as the product of device–vessel–patient interaction rather than a material-specific phenomenon.

### 8.4. Linking Imaging to Clinical Outcomes

Uncovered struts, malapposition, excessive neointimal growth, and neoatherosclerosis are biologically plausible substrates for thrombosis or restenosis, but the magnitude of clinical risk depends on prevalence, spatial extent, procedural context, antiplatelet therapy, and patient factors. Device development should avoid assuming that an imaging improvement necessarily predicts fewer myocardial infarctions or revascularizations.

A robust translational program ideally combines mechanistic assays, large-animal healing studies, intravascular imaging, and adequately powered clinical trials. This layered approach is particularly important for bioactive coatings and resorbable technologies, where intermediate biological advantages may not compensate for mechanical or procedural disadvantages. The experience with first-generation BRS is a clear example [28].

### 8.5. Clinical Endpoints as the Final Test of Material Innovation

The ultimate value of a biomaterial is determined by patient outcomes in the setting where the device will be used. Restenosis, target-lesion revascularization, stent thrombosis, myocardial infarction, and target-lesion failure integrate multiple biological and procedural mechanisms. Continued late event accrual after DES implantation [4] provides a rationale for innovation, but new technologies should be compared with contemporary rather than historical benchmarks.

This requirement is particularly important when engineering rationale is compelling. Polymer degradation, scaffold resorption, biomimetic coatings, or absence of an implant may each be biologically attractive. Their value becomes clinically meaningful only when the complete treatment strategy maintains or improves safety and efficacy.

## 9. Future Directions: Multifunctional and Healing-Responsive Coronary Biomaterials

### 9.1. From Single-Function Devices to Multifunctional Platforms

Most established coronary devices were optimized around a dominant function: mechanical support, suppression of neointimal proliferation, or transient drug transfer. Future platforms are increasingly conceived as multifunctional systems that coordinate several aspects of healing simultaneously. The ideal device would minimize platelet activation and chronic inflammation, control VSMC proliferation, promote functional endothelial recovery, and—where appropriate—disappear after its role is complete.

This ambition is technically demanding because biological targets can conflict. Potent antiproliferative therapy may delay endothelial repair, highly adhesive surfaces may promote both endothelial and smooth muscle cell attachment, and accelerated degradation may compromise radial strength. Multifunctionality therefore requires temporal as well as molecular control.

### 9.2. Smart and Responsive Drug Delivery

Responsive delivery systems seek to release drugs according to local biological signals rather than a fixed preset schedule. Candidate triggers include reactive oxygen species (ROS), pH, enzymes, inflammatory mediators, shear stress, ultrasound, or externally applied energy. In cardiovascular nanomedicine, stimuli-responsive polymers have been used experimentally to target inflammatory and atherosclerotic microenvironments [43,44].

For PCI devices, this concept could theoretically enable high local therapy during periods of thrombosis or inflammation while reducing unnecessary exposure during recovery. Smart balloon concepts are also being explored to improve transfer efficiency and reduce drug loss. At present, these technologies remain largely preclinical or early translational and should not be presented as established coronary therapies. Key barriers include trigger specificity, dose reproducibility, manufacturing scale, long-term stability, and regulatory complexity.

### 9.3. Next-Generation Local Therapeutics

The pharmacological repertoire of vascular devices may expand beyond conventional mTOR inhibitors and paclitaxel. Contemporary reviews describe emerging antiproliferative agents, nanocarrier systems, and more sophisticated local delivery approaches intended to improve tissue selectivity [18]. Potential future payloads include anti-inflammatory molecules, nucleic-acid-based therapies, pro-endothelial signals, or combinations delivered sequentially.

A particularly attractive concept is spatial selectivity: suppressing VSMC proliferation within the injured arterial wall while preserving or stimulating luminal endothelial recovery. Achieving this separation remains a major challenge because both cell types are exposed to the same local device environment. Layered coatings, directional release, cell-selective ligands, and temporally staged delivery are potential solutions.

### 9.4. Precision Biomaterials and Imaging-Guided Selection

No single biomaterial strategy is likely to be optimal for all coronary lesions. A calcified ostial lesion requiring substantial radial strength differs fundamentally from focal ISR, a small-vessel de novo lesion, or a long diffuse lesion in a patient at high bleeding risk. Future device selection may therefore become more explicitly phenotype driven.

Intravascular imaging can contribute by defining vessel size, plaque morphology, calcification, lesion preparation, stent expansion, and mechanisms of failure. Clinical variables—including bleeding risk, diabetes, acute coronary syndrome, thrombogenicity, and expected adherence to antiplatelet therapy—may further influence the preferred balance between mechanical support and implant burden. Figure 4 frames this as a transition from a single-function device toward precision coronary therapy. This remains a conceptual direction rather than a validated biomaterial-selection algorithm.

### 9.5. Manufacturing, Regulation, and Reproducibility

The complexity of future materials creates nonbiological barriers to translation. Multifunctional coatings must survive manufacturing, sterilization, storage, crimping, catheter tracking, balloon expansion, and prolonged exposure to blood. Nanoscale or biologically active surfaces may be particularly sensitive to changes in processing. Reproducibility across batches must be demonstrated at a level compatible with large-scale device production.

Regulatory evaluation will likewise become more complex when a device combines structural, pharmaceutical, and biologically active functions. Characterization will need to address mechanical integrity, coating chemistry, release kinetics, degradation products, particle generation, systemic exposure, immunogenicity, and long-term tissue response. Translational success will therefore depend as much on manufacturability and standardized testing as on biological novelty.

### 9.6. Evidence Standards for the Next Generation

A major lesson from coronary device history is that plausible mechanistic advantages can fail to translate into better outcomes. Future biomaterials should therefore be developed within an evidence framework that connects bench measurements to clinically interpretable endpoints. Early studies should define mechanism and durability; large-animal studies should assess healing under realistic implantation conditions; imaging should characterize structural response; and randomized trials should compare the complete treatment strategy with contemporary standards.

This staged approach may slow the apparent pace of innovation but reduces the risk of overinterpreting attractive surrogate findings. The objective is not simply to create a more sophisticated material, but to create a reliably manufacturable and clinically beneficial coronary therapy.

### 9.7. Economic and Access Considerations in Clinical Translation

Clinical translation is also constrained by economics. Acquisition prices vary substantially by country, purchasing contract, reimbursement system, and device generation, so universal unit-cost comparisons are not robust. Contemporary metallic DESs generally benefit from mature manufacturing, procedural familiarity, and broad supply, whereas specialized DCB formulations, bioresorbable scaffolds, and multifunctional bioactive platforms may carry higher or less predictable acquisition costs because of proprietary coatings, more complex processing, quality-control requirements, and smaller production scale. Total episode cost also depends on lesion-preparation devices, intravascular imaging, procedure time, bailout stenting, antiplatelet strategy, complications, and repeat revascularization; acquisition price alone therefore provides an incomplete measure.

Formal cost-effectiveness evidence remains limited and geographically dependent. A prespecified economic analysis of BASKET-SMALL 2 found only small differences between a DCB and DES in costs and quality-adjusted survival, with conclusions sensitive to comparator and time horizon [45]. This illustrates why economic claims should be linked to a defined indication and health system rather than generalized to a device class. For next-generation multifunctional or bioresorbable technologies, added manufacturing complexity will need to be justified by clinically meaningful reductions in repeat procedures, complications, or treatment duration. Where robust cost-effectiveness data are absent, this uncertainty should be reported explicitly as part of the translational evidence gap.

**Table 6:** Indicative economic and access considerations across coronary biomaterial platforms.

Platform	Indicative Acquisition Cost *	Major Procedural Cost Drivers	Cost-Effectiveness/Downstream Implications	Manufacturing/Access
Contemporary metallic DES	Moderate/mature market	Stent number; imaging; lesion preparation; optimization	Broadest evidence base; low restenosis/repeat PCI when well deployed; economic results remain health-system specific	Large-scale manufacturing and broad availability
Polymer-free DES/DCS	Moderate to high	Device price; short-DAPT pathway; imaging/optimization	Limited and device-specific; potential value in selected HBR pathways but no class-wide economic advantage	Specialized coating/reservoir manufacturing
DCB	Moderate to high; device-dependent	Lesion preparation; specialty balloons; imaging; bailout stenting	BASKET-SMALL 2 analysis showed small cost/QALY differences and sensitivity to comparator/time horizon [45]	Carrier/coating technology and reimbursement strongly influence access
PLLA bioresorbable scaffold	Historically high	Imaging; meticulous sizing/implantation; longer procedure time	Insufficient evidence for routine cost-effectiveness; theoretical long-term offsets were not established for first-generation PLLA platforms	Complex manufacture; prior market withdrawal limits access
Mg/Fe/Zn bioresorbable platforms	High/uncertain	Imaging; device-specific implantation; surveillance during early adoption	Sparse; no mature cross-platform economic evidence; downstream value requires durable comparative benefit	Alloy processing, corrosion control, coating, and regulatory testing add complexity
Bioactive/multifunctional surfaces	Unknown; likely high initially	Specialized manufacturing; functional testing; storage/sterilization requirements	No robust coronary cost-effectiveness evidence; clinical offsets remain unproven	Scale-up, reproducibility, stability, and regulatory complexity are major access determinants

* Cost categories are qualitative and relative because device prices vary substantially across countries, contracts, and reimbursement systems.

## 10. Conclusions

Coronary biomaterial performance is determined by the interaction among device architecture, material chemistry, local pharmacology, vascular biology, procedure, and patient context. A healing-centered framework therefore requires more than demonstrating thin struts, polymer disappearance, drug transfer, or OCT coverage. The relevant question is whether the complete strategy produces durable, functionally compatible vascular repair and clinically meaningful benefit compared with contemporary standards.

Five priorities emerge from this review. First, new devices should pair structural endpoints with functional endothelial and thrombogenicity measures rather than equating coverage with recovery. Second, mechanistic claims should be supported by primary evidence linking material chemistry to specific molecular and cellular pathways, including endothelial barrier/NO signaling and inflammatory or macrophage responses. Third, clinical conclusions should remain device-, formulation-, lesion-, and procedural-strategy-specific; results from one DCB, PLLA scaffold, or coating should not be generalized to an entire class. Fourth, future trials should integrate mechanistic testing, realistic post-deployment durability, intravascular imaging, randomized clinical endpoints, and health-economic assessment. Fifth, multifunctional and resorbable platforms should demonstrate scalable manufacturing and comparative effectiveness against best-in-class contemporary DESs before broad adoption. The next advance in coronary biomaterials will therefore be defined not by technological complexity alone, but by reproducible control of healing that translates into patient benefit and accessible care.

## Data Availability

No new data were created or analyzed in this study. Data sharing is not applicable to this article.
